# Monitoring and evaluating capacity building activities in low and middle income countries: challenges and opportunities

**DOI:** 10.1017/gmh.2016.24

**Published:** 2016-10-21

**Authors:** M. Schneider, T. van de Water, R. Araya, B. B. Bonini, D. J. Pilowsky, C. Pratt, L. Price, G. Rojas, S. Seedat, M. Sharma, E. Susser

**Affiliations:** 1 University of Cape Town, South Africa; 2 Stellenbosch University, South Africa; 3 London School of Hygiene and Tropical Medicine, UK; 4 University of São Paulo, São Paulo, Brazil; 5 Columbia University, New York, USA; 6 National Institute of Mental Health, National Institutes of Health, MD, USA; 7 University of Chile, Santiago, Chile; 8 Public Health Foundation of India, Delhi, India

**Keywords:** Capacity building, low and middle income countries, mental health, monitoring and evaluation, research, teaching and learning

## Abstract

**Background:**

Lower and middle income countries (LMICs) are home to >80% of the global population, but mental health researchers and LMIC investigator led publications are concentrated in 10% of LMICs. Increasing research and research outputs, such as in the form of peer reviewed publications, require increased capacity building (CB) opportunities in LMICs. The National Institute of Mental Health (NIMH) initiative, Collaborative Hubs for International Research on Mental Health reaches across five regional ‘hubs’ established in LMICs, to provide training and support for emerging researchers through hub-specific CB activities. This paper describes the range of CB activities, the process of monitoring, and the early outcomes of CB activities conducted by the five research hubs.

**Methods:**

The indicators used to describe the nature, the monitoring, and the early outcomes of CB activities were developed collectively by the members of an inter-hub CB workgroup representing all five hubs. These indicators included but were not limited to courses, publications, and grants.

**Results:**

Results for all indicators demonstrate a wide range of feasible CB activities. The five hubs were successful in providing at least one and the majority several courses; 13 CB recipient-led articles were accepted for publication; and nine grant applications were successful.

**Conclusions:**

The hubs were successful in providing CB recipients with a wide range of CB activities. The challenge remains to ensure ongoing CB of mental health researchers in LMICs, and in particular, to sustain the CB efforts of the five hubs after the termination of NIMH funding.

## Background

Capacity building (CB) in mental health is a strategy to improve mental health systems, services, and research in low- and middle-income countries (LMICs). CB will contribute towards reducing the treatment gap in these countries (Patel *et al*. 2011; Tol *et al*. 2011; Thornicroft *et al*. 2012). The need for CB in LMICs is underscored by the burden of mental, neurological, and substance use disorders (Steel *et al*. 2014; Global Burden of Disease Collaborators, 2015), and the proportionately low representation of research publications from lead authors in LMICs in leading mental health journals (Patel & Kim, 2007; Sharan *et al*. 2007).

The 2013 Global Burden of Disease study (Global Burden of Disease Collaborators, 2015) highlights mental disorders as leading causes of years lost to disability. This burden falls heavily on LMICs – home to 85% of the world's population (Sharan *et al*. 2009). Yet the research output regarding mental health is comparatively low for LIMCs. Only 6% of publications on ISI Web of Science (from 1992 to 2001) (Saxena *et al*. 2006) and 3.8% of publications in six prominent psychiatric journals from 2002 to 2004 (unchanged from 1996 to 1998 results) (Patel & Kim, 2007) were from lead authors in LMICs. Reasons for limited published mental health research and limited mental health services in LMICs include: (i) high levels of stigma towards mental disorders, (ii) lack of sufficient and/or appropriate professional development opportunities in mental health research, (iii) limited funding for and culture of research, (iv) few skilled researchers, and (v) few collaborative endeavours (Sharan *et al*. 2007; Sweetland *et al*. 2014).

CB initiatives help to address these barriers through strategies such as (i) south-north and south–south collaborations (Sweetland *et al*. 2014), (ii) harnessing collaborative initiatives to produce high quality research data, and (iii) using twenty-first century technology that makes information and distance learning more accessible than ever before. While it is important to develop CB initiatives, it is also important to incorporate a monitoring and evaluation (M & E) component to ensure that these initiatives achieve their CB aims.

The National Institute of Mental Health (NIMH) has funded five collaborative hubs for mental health research – two in Latin America – Regional Network for Mental Health Research in Latin America [RedeAmericas (Susser *et al*. 2012] and Latin American Treatment and Innovation Network in Mental Health (Latin-MH), two in sub-Saharan Africa – Partnership for Mental Health Development in sub-Saharan Africa (PaM-D) and Africa Focus on Intervention Research for Mental Health (AFFIRM) (Lund *et al*. 2015) and the South Asian Hub for Advocacy, Research and Education on Mental Health (SHARE) hub in south Asia. The aim of these hubs is to develop research on task sharing interventions for mental health, develop researcher networks, and build capacity in mental health research in their respective regions. The CB strategies developed by the five hubs has been described in a sister paper (Pilowsky *et al*. 2016) and in the specific hub papers in this special issue. As suggested by Sharan *et al*. (2007), mapping and monitoring CB activities should be an important component of CB strategies. This paper aims to describe and monitor the outcomes of CB activities for research conducted by five research hubs to showcase the range of feasible CB activities and provide initial indications for successful strategies.

## Methods

A basic M & E framework was developed from which the common indicators were extracted for comparison of the CB activities and outcomes across five hubs.

### Development of the M & E framework

Firstly, every hub compiled a list of their CB opportunities. This list described the content, target audience, application process, cost, attendance, and outcomes for each activity. Secondly, each hub set out their main indicators (including the number of student-initiated publications, number of courses and workshops with attendance and completion rates, and grant applications submitted) to be used for M & E the outcomes from these CB activities. The second author evaluated these lists to identify the common indicators, which were subsequently set into standardized forms for data capturing within hubs on an ongoing basis. Table 1 illustrates the two categories for the common indicators used for the cross-hub analysis presented in this paper, namely (i) CB activities and (ii) monitoring of outputs.

**Table 1.** Comprehensive list of the indicators used for Monitoring and Evaluating the capacity building (CB) strategies of the five collaborative hubs

Category	Indicators
CB activities	
Intra-hub courses and workshop^a^	No of courses, No of participants registered and completing the course/workshop
Inter-hub courses and workshops	No of courses, No of participants registered and completing the course/workshop
Monitoring of outputs	
Publications by fellows, mentees or awardees	Local inter hub, international inter hub, local intra hub, international intra hub, local publications, international publications first authored by the fellows/awardees/students
Grants submitted by fellows, mentees or awardees	Local grant applications submitted, successful/unsuccessful local grant applications, international grant applications submitted, successful/unsuccessful international grant applications
Tracking of fellows, mentees or awardees	Career pathways and involvement in further degree training, grant writing, research and publications

a Intra-hub activities are usually multi-country in focus but within one hub, while inter-hub activities involving more than one hub.

### Description of common indicators

CB activities were mostly centred around workshops and courses. Some of these CB activities were available only to participants within a hub (intra-hub) while others were available for participants across the hubs (inter-hub).

Apart from immediate course evaluation surveys examining the subjective experience and perceived benefit of the CB activity, hubs also monitored subsequent outputs of participants. For example, in the PAM-D hub former CB participants were regularly followed up via email to monitor their publication outputs, grant applications, and career progression, while for the AFFIRM hub a brief survey was sent to MPhil graduates towards the end of 2015.

Although some of the hubs obtained Institutional Review Board (IRB) approval for the overall hub project, others only obtained IRB approval for the randomized controlled trials (RCT) run within the hub. No IRB approval was obtained specifically for the CB activities from any of the hubs as this did not involve research with human subjects.

## Results

The results from the M & E exercise for the five hubs combined are divided into two sections: (i) CB activities (attendance and completion rates for both intra-hub and inter-hub activities) and (ii) M & E indicators (publications, grant applications, and career tracking). As the paper describes the trends across hubs, the detail is limited to an overview with additional detail available in the other hub-specific papers in this special issue.

### CB activities

#### Intra-hub courses and workshops

Table 2 sets out the individual courses for each hub and how many fellows or awardees were selected and completed the training. As the PaM-D and Latin-MH hubs started later, they have completed fewer activities than the other three hubs.

**Table 2.** Attendance and completion for capacity building (CB) activities of the five Collaborative Hubs for International Research on Mental Health

Africa Focus on Intervention Research for Mental Health (AFFIRM) (2011–2015)				
Sponsored activity	Type of CB activity	Contents	Participation	Entry criteria
MPhil Public Health (1 year, extendable to 2 years)	Masters: 3 weeks of face to face teaching in Cape Town in January; remainder through electronic supervision. Monthly webinars are having positive effect on completion time	Training in public mental health research. Working closely with supervisor on a proposal, structured literature review, data collection and write up of analysis into a thesis	25 enrolled: 8 graduated 2 dropped out 1 passed away 14 ongoing	Hons level qualification and already involved in mental health related services and/or research in home country; recommended by in-country AFFIRM team
Randomized controlled trials (RCT) in mental health course	Short courses presented face to face over 5 days in Cape Town, South Africa; Addis Ababa, Ethiopia	Topics responded to expressed needs in region and positive comments from course participants as to the benefits of these short courses. training in a specific topic within public mental health; e.g. RCTs	30 complete (100%)	N/A depends on course topic
RCT in mental health course			30 complete (100%)	
Operations research in mental health	2 day course, Blantyre, Malawi		40 complete (100%)	
Ph.D. candidates throughout AFFIRM duration	4 Ph.D.s are linked to two ongoing RCTs in AFFIRM countries (Ph.D. face to face)	South Africa (3) and Ethiopia (1); 1 Ph.D. is linked to the inter-hub Shared Project in Ethiopia. Registering for Ph.D. using the AFFIRM projects as the basis for the Ph.D. topic	5 enrolled: 5 ongoing	Masters level completed; requirements as per the registering university
Latin-Mental Health (MH) (2013–2015)				
Postdoctoral fellowships (12 months)	12 months of research work within the Latin-MH Hub; Face to face post-doctoral fellowship for 3 fellows from Latin-MH Hub countries	To produce scientific output and to help developing further research proposals. To be involved in key positions for the coordination of the hub's activities. To develop a post-doctoral research within the goals of the project as well as to develop skills in the coordination of the hub activities	6 enrolled: 2 completed 4 ongoing	Ph.D., research experience, interest in global mental health, likely to make an impact on mental health research and services in their country
Junior (12 month graduate fellowships with possibility of extending)	The fellows will receive joint mentorship from a local and an international senior researcher involved with the hub's activities (face to face)	To develop their own research within the goals of the hub. Some fixed activities run by the hub. There is no fixed curriculum, as they will depend on the level of experience of the fellow as well as the training approach provide by each of the participating institutions	2 enrolled: 2 ongoing	Graduation students/mental health workers interested in global mental health research. Inserted in a graduation program in one of the Latin-MH partner universities then approved by both PIs to be included as a junior fellow
RCT fellows (from mid 2015/2016–end of trial)	Young researchers who are invited to work face to face in the main research proposal funded by the hub	To work in the RCT that will happen in Brazil and Peru in the next years; and to write their own papers using the trial data collected in the last year of this grant. To work in the RCT and to develop research in global mental health	1 enrolled: 1 ongoing	Masters or Ph.D. students. To be a grad student in one of the two universities partners of the hub (USP and Cayetano Heredia)
Symposium (June 2015)	1 panel 2 workshops Face to face presentations made available online afterward	To develop workshops in different subjects that are led by the senior members of the hub and to identify possible researchers to be fellows of the hub. To participate in a full day activity about RCT designs and Mobile Technological intervention, as well as participate in the discussion of global mental health research and opportunities in different countries	36 completed (100%) 24 completed (100%)	Local young researchers interested in Global Mental Health providing a statement on why they are interested in this activity and a brief resume (professor, researcher or health worker)
Summer school (July 2016) 1 week	Face to face presentations made available online afterward	To prepare the students to understand about RCTs and other topics related with the hub's research as also as prepare them to work with the hub. Classes about the main research topics: methodological tools for the development of mental health research in the primary health context	Data unavailable	To be a grad student and be interested in working with the Latin-MH hub as a RCT fellow
Partnership for Mental Health Development (PaM-D) (2013–2015)				
Mental Health Leadership and Advocacy Programme (2 weeks in Ibadan, Nigeria, June 2013)	Face to face course to build expertise in using evidence for policy formulation, critical review of the evidence, consultative decision-making, operational issues such as workforce needs assessment, how to generate and support the workforce and other issues	Course comprises individual and group work, Mental Health systems in low- and middle-income countries (LMICs), Mental Health Service Development, Mental health legislation, Health system financing, Developing feasible and effective health policy, Mental health promotion, Monitoring and evaluation of health systems	29 completed (100%)	Mental health professionals and senior policy makers
Biostatistics introductory and intermediate courses (5 days, Cape Town, 2014)	Face to face workshop with biostatisticians	To discriminate between different types of data (e.g. continuous, categorical, binary); understand statistical terms such as sample, population, probability distribution, *p* value; work with descriptive data and display data in tables and diagrams (descriptive statistics); calculate and interpret confidence intervals; carry out and interpret the results of hypothesis testing on continuous and categorical data; understand different tests of association; calculate sample size and understand power considerations; have a basic understanding of how to use statistical software, like STATA, to analyse and present their data	22 completed (100%)	Mental health care practitioners (psychiatrists, psychologists, registrars, social workers) with a Master's degree of equivalent
Small grants applications (February 2015 ongoing)	To provide mentorship via email and grant funding for early career researchers	To budget and manage research funds through writing regular feedback reports	4 enrolled: 4 ongoing	3 emerging mental health researchers with a research proposal for a masters/Ph.D.
Scientific writing workshop (4 days in Johannesburg, SA, 2015)	3 emerging mental health researchers identified by country leader invited to attend practical face to face writing workshop	To train emerging researchers in the basic skills of article writing in order to get published in higher impact factor journals. Apply the process of taking a research idea, phrasing it into a research question and turning it into a hypothesis. Overcome the obstacles to high quality research in low income settings. Identify best research designs and methods. Choose data analysis strategies to match the hypothesis. Apply writing tips to an abstract, introduction, and discussion. Identify peer-reviewed journals most suitable for their research. Knowledgably prepare for a poster or oral presentation. Identify resources and collaborative support in the research process	13 enrolled 11 completed 2 participants could not attend due to Ebola related travel restrictions	Masters or equivalent, emerging mental health researcher with limited publication experience
Early Career Fellowship Exchange (from year 2)	2 weeks intensive face-to-face opportunity with ongoing support	Building expertise in Cochrane systematic reviews, collaborative care interventions	1 completed (100%)	Nigerian fellow spent 2 weeks in the UK being mentored by a consultant on the Hub on writing a systematic review
RedeAmericas (2011–2015)				
Sponsored activity	Type of CB activity	Contents	Participation	Entry criteria
2 year MSc/M Public Health	Masters over 2 years	To extend opportunities and support for students in a MPH or equivalent academic program that teaches concepts and methods applicable to community-based mental health research	6 completed (100%)	Must meet university requirements for admission
Ph.D. candidates	Ph.D.	To develop their research interests and work on their theses as a platform for independent research in community-based mental health in their countries of origin	8 enrolled: 3 completed 5 ongoing	Must meet university requirements for admission
Epidemiology and Population Health Summer institute, Columbia University	There are 4 weeks of courses offered every year in June (introductory and advanced). Each course is a half-day (4 h in am or pm) for 1 week (20 h in total) and provided face to face whereafter the presentations are made available online	Offer short, focused epidemiology and population health courses at a world class university. There are foundational courses, skill building courses, and courses on specific health domains. There are 28 courses offered for the 2013 summer institute	5 completed (100%)	Students, investigators and scholars from the health and social sciences, public health practitioners, clinicians, and industry professionals interested in deepening their knowledge of population health. (See listings on https://www.cuepisummer.org/courses)
Epiville [online learning tool developed specifically for Principles of Epidemiology (P6400)]	Epiville is a set of interactive web-based exercises created by faculty in the Department of Epidemiology and produced by the Center for New Media Teaching and Learning at Columbia University. No specified time points (one can start and stop whenever). There are 10 modules and each is designed to last approx. 1 week. Best used to complement an in-person epidemiology course	The primary goal of Epiville is to provide an enhanced web-based learning environment so that students can most efficiently master the main principles of the course. We believe that the tools employed in Epiville – instantaneous answers to multiple-choice questions, use of interactive maps and visuals, and open-ended questions for discussion in seminar meetings – will contribute to improving your capacity to collect and analyze epidemiologic data and, ultimately, to your ability to carry out independent work in the field. See http://epiville.ccnmtl.columbia.edu/modules.html for information	11 completed (100%)	Anyone who understands English and meets the computer requirements. Open to anyone who desires an introduction to epidemiology
University of Chile Summer Institute School of Public Health	2 weeks in January 2014: Classes are half day (4 h) for 1 week (face to face). A few courses are full day	To offer wide-ranging, skill- and knowledge-based 1 week courses on diverse topics in public mental health. This enhances the professional development of students in public mental health. Two RedeAmericas investigators from the USA travelled to Santiago to teach courses	12 completed (100%)	From students just beginning their public health study to established professionals looking to learn the latest technologies and applications (aka, varied). Depending on each course (some require no former work, others require masters level knowledge). Courses are in Spanish, unless otherwise noted
International conferences	Awardees attended as many as 6 international conferences, were leading organizers of 3 of these conferences, and presented abstracts and/or led round table discussions	To enhance opportunity for collaboration; to improve international and regional mental health networks; to share experiences, challenges, and progress; and to learn from indepth presentations by world-renowned speakers on relevant concepts and methods	9 completed (100%)	Awardees used their funds to attend conferences relevant to their research interests. They also presented their own research at these conferences. Several were leading organizers and established a new international conference in the region
Grant applications	To support the development and submission of grant proposals to funders	Awardees worked closely with mentors on each step of developing a grant proposal, from the initial evaluation of the opportunity to fine-tuning the proposal and successful submission	Six participated in the development of 23 funded proposals. Number of proposals per participant ranged from 1 to 7. Completed (100%)	Six awardees are now PIs or major co-investigators on funded grants. Five awardees have been accepted into Ph.D. and DrPH programs since the start of the study and are continuing to work on grant preparation with their mentors
Guidance on career development and academic appointments	To support career development among early stage mental health researchers	Guidance on achieving professorial appointments at universities in the region to continue mental health research	11 completed (100%): 6 Asst Prof and 5 Academic Research Positions	Must meet university criteria
South Asian Hub for Advocacy, Research and Education on Mental Health (SHARE) (2012–2015)				
Sponsored activity	Type of CB activity	Contents	Participation	Entry criteria
Fellowships	1 year mentored research study undertaken by the awardee in the area of their interest	The focus of research study is chosen by the awardee, it is expected that the research study should focus on an issue related to the mental health treatment gap in South Asia	8 completed (100%)	Fellows should be associated with SHARE partner institutes; can show a career commitment to work in area of mental health research in South Asia
2 year Mental Health services in humanitarian context	A part time 2 year course that includes 4 onsite modules. Each module is 2 weeks long and is followed by online mentoring by the course faculty	The course content is divided across 4 modules: Qualitative assessments Intervention development and programme planning Instrument development, validation and use in baseline studies Evaluation and dissemination	8 completed (100%)	Awardees should be associated with SHARE partner institutes and working in humanitarian context in South Asia
Implementation Science distance learning course	This online course provides real time webinars for participants from all NIH hubs	The course aims to provide a systematic framework for implementation and evaluation of public health interventions. The course is divided in 6 modules. 1: Frameworks for successful implementation. 2: Designing for implementation. 3. Readying for implementation. 4. Implementing with quality. 5. Evaluating processes and outcomes. 6. Sustaining and improving change	16 enrolled: 12 completed 4 did not complete the course	Researchers and practitioners working in area of health/development with degree in health-related disciplines. 2 years (minimum) of work experience in healthcare/public health/development sector
Global mental health online lectures and forum	This online course is still in development together with RedeAmericas	The online library would have lectures on Principles and Practice of Global Mental Health and the regional or country specific examples. 9 Power point lectures have been uploaded under the Global Mental Health section of website	10 lectures uploaded: 34 (expandable)	Researchers/Practitioners/students interested in mental health
Studentships for short courses	Scholarships to early career researchers to participate in short courses in South Asia	Scholarships provided for these three courses: Leadership in mental health; Qualitative Methods for Health Research and Medical Discourse; Development and Evaluation of Complex Interventions	17 completed (100%)	Entry level researchers associated with SHARE network partners institutes

*Overview of CB activities*: All five hubs ran courses and workshops from the first year. Evident in Table 2, these varied extensively in time range and content, from full postgraduate degree courses to short face-to-face or online courses and mentorships. Where possible, presentations were made available online after the CB activity.

*Overall completion rates*: The completion rate for the courses across all hubs is high (ranging between 75% and 100%). By nature, short courses are more feasible to complete and demonstrated highest completion rates. Short courses were run by internationally recognized experts in the field. Although still high, courses with longer duration tended to have lower completion ratios than short courses. The high completion rates are not unexpected as the stringent nature of the selection process together with good funding (Table 2) ensured that participants were motivated and able to attend and complete the courses, and participants had access to support systems and mentoring through the allocation of course supervisors or mentors as evident by examples from different hubs.

*AFFIRM* runs the MPhil in Public Mental Health. This is a 2 year full time course providing supervision (primarily by the Stellenbosch University or University of Cape Town with in-country co-supervisors) to students from five sub-Saharan African countries. Most of the students are working full-time while completing their dissertation. This, coupled with various challenges in maintaining good contact between students and supervisors, has meant that a number of students have either not completed or taken longer than the 2 year funded period, which requires that they find their own funding for the final year. In addition, AFFIRM hosts Ph.D. students linked to the RCT and ran three short courses (see Table 2 and AFFIRM specific paper in this volume for further information).

*Latin-MH* provides national and international mentorships to its fellows. All fellows are supported in writing their own papers and to participate in the hub's publications. The young researchers, who are invited to work in the main project, have a ‘hands-on’ approach to activities related to the preparation and fieldwork of the hub's RCT. Some are also supported in writing applications for formal postgraduate training and grant applications. All fellows have mandatory activities provided by the hub that need to be developed during their fellowships and are monitored in their activities and their development. Postdoctoral fellows develop their own research within the main goals of the hub.

*PaM-D* provides mentoring and supervision through a number of CB activities. (1) The Mental Health Leadership and Advocacy Programme (mhLAP) targets future mental health leaders and advocates from five English speaking countries in West Africa (Nigeria, Ghana, Sierra Leone, Liberia and Gambia) as well as the other two hub countries, Kenya and South Africa. Overall, the course was positively received with participants indicating that they welcomed the content focus on mental health legislation, policy, advocacy, prevention and promotion of mental health, indices of disease burden, as well as interacting with mental health practitioners from across Africa. (2) Two biostatistics short courses (beginners and intermediate) spanning 5 days were held in Cape Town, South Africa in mid-2014 with four senior biostatisticians/statisticians facilitating the course and 22 early career researchers from the five hub countries in attendance. (3) In March 2015, PaM-D hosted a three and half day applied writing workshop in Johannesburg, South Africa. Participants found the workshop very beneficial overall, with practical writing exercises particularly useful. The workshop facilitator continues to mentor workshop participants even months afterward. (4) Finally, an early career fellow within the Hub undertook a 2-week research mentored attachment in February 2016 at the Institute of Psychiatry, Kings College London, to acquire skills in systematic review writing. A second fellow will be undertaking a similarly focused attachment at the University of Stellenbosch, Cape Town, South Africa in 2016.

*RedeAmericas* provides support for young mental health professionals from Latin America who have shown potential to become successful independent investigators in projects related to the mental health needs of the region. Each awardee has a personalized, flexible training program. Core elements in the training program for most awardees are summer courses at Columbia University and University of Chile, and a mentoring program that includes the assignment of two mentors – one from Latin America and one from Columbia University. In addition, the majority of awardees participate in international and regional conferences, many of them play key roles in organizing these conferences, and through such activities they develop a self-sustaining regional network (which also includes non-awardees with similar interests). For example, awardees were central to the organization of three regional conferences: a Latin American Congress of Public Health in Cordoba, Argentina; a Latin American Congress of Epidemiology in Medellin, Colombia; and a Latin American Congress on Autism Spectrum Disorders in Santiago, Chile. Awardees are encouraged to pursue higher degrees such as MPH or Ph.D., and then to take academic positions as Assistant and later Associate Professors, which enables them to pursue research careers in public mental health. The mentors facilitate the progress of the awardees through all these CB activities including the design of the awardees’ first research proposal.

*SHARE* senior researchers provide guidance and mentoring to junior researchers to develop a career in mental health research, three SHARE researchers have enrolled in Ph.D. programs and embedded their doctoral project in ongoing SHARE projects. One SHARE fellow has won a grant to scale up his fellowship study in Sri Lanka. The SHARE core team also provides support to researchers and fellows in disseminating their work to a larger audience. All SHARE fellows have presented the results of their studies at annual SHARE meetings. Some of them were supported to present their study at the Global Mental Health Summit (hosted by SHARE) held in India in November 2015.

#### Inter-hub courses and workshops

In addition to the hub-specific CB activities described above, a number of the CB activities were also available to other hubs. The supercourse currently being developed jointly by the SHARE and RedeAmericas hubs is openly available for all hubs to use on an ongoing basis (http://www.pitt.edu/~super1/GMHS/index.htm). Other CB activities had limited participation from other hubs due to a limited number of places available on the course (e.g. limited places on the MPhil in Public Mental Health and the Implementation Science distance learning course) and geographical location of the courses (e.g. biostatistics course in Cape Town by PaM-D and RCT courses by AFFIRM in Cape Town and Addis Ababa). The materials from many of the courses are available on the hub websites (e.g. RCT course materials on the AFFIRM website). The SHARE hub ran a 6 week online course on implementation science and invited two participants from each of the other hubs. Table 3 presents a matrix of the courses run by the different hubs available and attended by other hubs.

**Table 3.** Summary of interhub capacity building (CB) activities across the five hubs

Hub running the course Hubs attending	Africa Focus on Intervention Research for Mental Health (AFFIRM)	LATIN-Mental Health (MH)^a^	Partnership for Mental Health Development (PaM-D)	RedeAmericas	South Asian Hub for Advocacy, Research and Education on Mental Health (SHARE)	National Institute for Mental Health (NIMH)
AFFIRM			Beginners and intermediate Biostats course	Supercourse	Supercourse Implementation Science course	Webinars on various topics
LATIN-MH				Supercourse	Supercourse Implementation Science course	Webinars on various topics
PaM-D	5-day randomized controlled trails (RCT) courses – both in Cape Town and Addis Ababa			Supercourse	Supercourse Implementation Science course	Webinars on various topics
RedeAmericas				Supercourse	Supercourse Implementation Science course	Webinars on various topics
SHARE				Supercourse	Supercourse Implementation Science course	Webinars on various topics

a LATIN-MH is still at the early stages of the 5 year project and hence have little to report on as yet.

In addition to the CB activities run by the five collaborative hubs, the NIMH ran a number of webinars on topics identified by the hubs together in the regular CB workgroup teleconference meetings. The topics included complex interventions, presentations from researchers in the different hubs, and the range of funding opportunities available from the NIMH. These webinars were advertised widely by each hub and participation was open to all. Some challenges to participation were time zone differences and access to sufficient bandwidth to stream the webinar online.

One of the key benefits of inter-hub CB activities is the development and maintenance of a network of early and mid-career researchers in mental health across regions. Networks facilitate the development of collaborative projects and grant applications. Individual hub countries also provide opportunities for more localized networks to be developed, for example groups of students attending a course together.

*At a different level and not specifically monitored*, further CB opportunities are provided through each hub's annual meetings where all the hub countries come together and discuss the research being undertaken in the hubs. These annual meetings include CB activities or reports from fellows or students benefiting from them, or specific CB activities for the meeting participants. Participation in hub meeting discussions and presentations of the different intra-hub research activities contributes to CB of hub partners. The AFFIRM hub partners specifically commented on the benefit they received from. The AFFIRM hub partners specifically commented on the benefit they received from being part of the annual meeting discussions. The SHARE hub organizes a CB workshop with its annual meeting, including the SHARE researchers, fellows and public health practitioners working in the host country. So far, SHARE hub has organized such CB workshops on maternal mental health for local researchers in India, Bangladesh, and Sri Lanka.

### Monitoring of outcomes

While the number of courses run, attendance numbers and completion rates are important outputs of planned CB activities, it is important to consider other more medium to long-term outcomes from the overall CB programs. These include reviewing the number of publications generated by participants in the CB activities, number of new grant applications submitted, and career pathways forged. Longer-term outcomes would include the reduction in the mental health treatment gap and sustainability of network within countries, regions and at a global level. This paper reviews the more short- and medium term outcomes of the CB activities of the five hubs. The publication describing the individual hub CB activities provides further information on individual hubs’ monitoring approaches (Pilowsky *et al*. 2016), as do the individual hub-specific papers in this special issue. The monitoring indicators implemented by each hub are presented in Table 4. All hubs prioritized publications, short course attendance, pre/post-course evaluations, and tracking of students as important indicators of outcomes. The other indicators (degree completion, participation in hub activities and progress reports for small grants were focused on hub-specific CB activities and were thus limited to the relevant hubs. Tracking of students has started but this is an ongoing and more long-term monitoring indicator that has not yielded much information as of early 2016.

**Table 4.** Monitoring indicators selected by each hub

	Africa Focus on Intervention Research for Mental Health (AFFIRM)	LATIN-Mental Health (MH)	Partnership for Mental Health Development (PaM-D)	RedeAmericas	South Asian Hub for Advocacy, Research and Education on Mental Health (SHARE)
Indicator					
Degree completion and granted	X			X	X
Attendance and completion rates for courses/workshops	X	X	X	X	X
No of publications (completed, in progress)	X (1, 5)	X (0, 3)	X (1, 3)	X (9, multiple)	X (2, multiple)
Participation in hub activities		X		X	
Pre- and/or post training surveys	X	X	X	X	X
Tracking of students	X	X	X	X	X
Project and budget progress reports for small grant awards			X	X	

#### Grants

Across all the hubs two local and six international grant applications were submitted, with SHARE and RedeAmericas focussing specifically on grant application as a CB activity. The SHARE hub has had three grant applications funded, two of which were developed with the assistance of CB fellows and one was prepared with a fellow as the lead. Further details can be obtained at the SHARE website (http://www.sharementalhealth.org).

RedeAmericas career awardees have had some success in receiving grant funding. Overall, six of the 13 awardees have received local grants as the principal investigator or co-investigator on a study since their training in the RedeAmericas program. Another awardee has recently submitted an application for a local grant to develop an RCT study and is waiting for approval and funding. For the other hubs grant proposals are anticipated as a medium to long-term outcome of increasing skills in mental health research.

#### Publications

All hubs used publication counts as a monitoring indicator. For this indicator we included only those publications where CB participants were lead authors. Across all hubs, a total of 13 international publications from individual hub students or trainees as lead authors have been accepted for publication. A number of further publications have been submitted for review or are in preparation (Table 4). The individual hub counts are as follows: (i) AFFIRM has one publication by a graduate from the MPhil programme (Udedi *et al*. 2013) and around four to five others in preparation; (ii) LATIN-MH – has three publications in preparation at the moment; (iii) PaM-D – one peer reviewed publication (Nortje *et al*. 2016), three under journal review and a number more in preparation; (iv) RedeAmericas has nine awardee-led peer reviewed publications with a number led by awardees in preparation; (v) SHARE has two publications led by fellows (Siriwardhana *et al*. 2013; Adhikari *et al*. 2015) and a number in preparation.

In RedeAmericas, there has been a strong focus on publication and each of the 13 awardees contributed valuable insight and information as authors on peer-reviewed publications. Throughout their time in RedeAmericas, they have collectively contributed to 61 articles published in local journals and 33 articles in international journals. Nine of these awardees have been listed as first author on a peer-reviewed publication. Most of their work has been published in local journals such as the *Revista Argentina de Psiquiatria*, the *Jornal Brasileiro de Psiquitria*, the *Cadernos Saude Coletiva*, and the *Revista de Salud Publica*. Three awardees have published peer-reviewed articles as first author in international journals including the *Journal of Clinical Psychopharmacology*, the *Journal of Affective Disorders, Frontiers in Psychiatry*, and the *Archives of Psychiatry and Psychotherapy*.

#### Tracking of students

This indicator considered career pathways and involvement in further degree training, grant writing, research, and publications by participants in the major CB activities such as mentorships and degree courses. The hubs reviewed dissemination of research information from students (e.g. in publications, blogs, or other formats) and tracking of career pathways on completion of their courses. For the AFFIRM hub, one of the earlier graduates from the MPhil has subsequently moved into a senior position in the Ministry of Health in his country. A number of AFFIRM MPhil students and graduates contributed to a blog developed as a lead-up to a panel session on the contribution of students to Global Mental Health presented at the International Mental Health Congress held in April 2015 in Lille, France (see https://storify.com/MHIN/studygmh). This blog was hosted by the Mental Health Innovation Network (MHIN).

The PAM-D hub requests regular progress and feedback from small grant awardees. In addition, the writing workshop candidates are closely followed up via email to determine the publication outputs upon completion of the course.

## Discussion

The combination of the treatment gap, limited skills, lack of research, and few publications emanating from LMIC researchers in the field of mental health in LMICs requires a clear strategy to rectify the situation. Thornicroft *et al*. (2012) advocate addressing this through an increase in research funding, building capacity in mental health research through networks and collaborations, and fostering a culture of and incentives for mental health.

The importance of building partnerships through collaboration, networks, and mentorships between internationally recognized mental health research centres and developing researchers in LMICs is an important component of changing the visibility of mental health in LMICs (Fricchione *et al*. 2012; Sweetland *et al*. 2014). The allocation of a Latin American and US-based mentor for the RedeAmericas awardees is a good example of this, as was the use of internationally recognized mental health researchers in the numerous short courses run by the different hubs.

The CB strategies and related activities have as their aim the reduction of the treatment gap and an increase in mental health research in LMICs. All the hubs included aspects of research skills development and mentorship from both local and internationally recognized mentors, and included efforts to build sustainable networks. These networks were developed through regular meetings, webinars, and conference attendance for fellows, mentees or awardees.

The interest shown in all the hubs to participate in these CB activities (e.g. high number of applicants and good completion rates) attest to the growing interest in mental health research and in building knowledge and skills. These CB activities are well appreciated. However, monitoring of the impact of these CB activities is a medium to long-term endeavour and requires a ‘robust set of metrics’ (Cottler *et al*. 2015). As participants attend numerous CB initiatives it is challenging to identify which type of CB activity yields the most beneficial research output. Furthermore, over time we can consider the impact of different levels of research infrastructure or publication culture on outputs when comparing middle and low income countries and also determine whether the hubs made a difference to research productivity in the LMICs identified for inclusion. For example, repeats of the reviews cited at the start of this paper on the number of publication emanating from lead authors from LMICs (Saxena *et al*. 2006; Patel & Kim, 2007) could indicate changes over time that could be attributed to all CB strategies including of the hubs described in this paper.

Another medium to long-term challenge is expanding the research-training networks of the Hubs beyond individual researchers, groups, and academic institutions to include non-governmental organizations and policymakers. Effective monitoring of the success (quality) and sustainability of the mentorship of young trainees remains another challenge. In addition, other metrics such as memberships of professional societies, further degrees awarded, faculty appointments, and cross-cultural competencies gained should be specifically measured and monitored (Cottler *et al*. 2015).

The cost of the CB activities is high as it often requires much travelling, course registration fees, and costs of carrying out actual data collection. This does bring into question the sustainability of ongoing CB activities. But there are initiatives to fund ongoing CB and ensuring sustainability of these budding networks. One such initiative is the 5-year Welcome Trust funded CB project entitled Africa Mental Health Research Initiative (AMARI), which addresses CB in four countries (Ethiopia, Malawi, Zimbabwe and South Africa) at masters, doctoral, and post-doctoral level. The MHIN (see www.mhinnovation.net) provides a platform for blogs and dissemination of mental health research findings and comments from mental health researchers, practitioners, and people with mental disorders at a global level. Currently the MHIN has a global reach and is developing an African office (MHIN Africa) targeting this region.

Sustainability of CB initiatives will take time to develop with immediate costs being high until a critical mass of skills and research experience has been reached within individual countries and regions to sustain ongoing locally led development of CB, research, and grant applications.

In conclusion, the CB strategies and activities of the five Collaborative Hubs for International Research on Mental Health are proving successful in building research capacity within countries where it is most needed. The ongoing impact and sustainability of these efforts will emerge in the next decade. If the success continues, the impact will ensure culturally relevant and appropriate research that will address local needs (Saraceno & Saxena, 2004; Saraceno *et al*. 2007; Thornicroft *et al*. 2012) and building an evidence base for global mental health by contributing different geographical and cultural perspectives. The challenge remains to ensure ongoing CB of mental health researchers in LMICs to sustain the efforts of the five hubs.
